# Human Resistin Is a Systemic Immune-Derived Proinflammatory Cytokine Targeting both Leukocytes and Adipocytes

**DOI:** 10.1371/journal.pone.0000031

**Published:** 2006-12-20

**Authors:** Ivan Nagaev, Maria Bokarewa, Andrej Tarkowski, Ulf Smith

**Affiliations:** 1 The Lundberg Laboratory for Diabetes Research, Department of Internal Medicine, The Sahlgrenska Academy at Göteborg University Göteborg, Sweden; 2 Department of Rheumatology and Inflammation Research, The Sahlgrenska Academy at Göteborg University Göteborg, Sweden; Centre de Regulació Genòmica, Spain

## Abstract

The characteristics of human resistin (RETN) are unclear and controversial despite intensive adipose-focused research. Its transcriptional and functional similarity with the murine myeloid-specific and CCAAT/enhancer binding protein epsilon (Cebpe)-dependent gene, resistin-like gamma (Retnlg), is unexplored. We examined the human CEBPE-regulatory pathway by unbiased reference and custom gene expression assays. Real-time RT-PCR analysis demonstrated lack of both the transcriptional factor CEBPE and RETN expression in adipose and muscle cells. In contrast, primary myelocytic samples revealed a concerted CEBPE-RETN transcription that was significantly elevated in inflammatory synoviocytes relative to intact peripheral blood mononuclear cells (PBMC). Mouse Cebpe and Retnlg were predictably expressed in macrophages, whereas Retn was abundant in adipocytes. Quite the opposite, a low and inconsistent RETN transcription was seen in some human white adipose tissue (WAT) biopsies without any relationship to body mass index, insulin sensitivity, or fat depot. However, in these cases, RETN was co-detected with CEBPE and the leukocyte-specific marker, EMR1, indicating the presence of inflammatory cells and their possible resistin-mediated effect on adipocytes. Indeed, addition of human resistin to WAT in culture induced, like in PBMC, the inflammatory cytokines IL6, IL8 and TNF. Importantly, the expression of the adipose-specific markers CEBPA, FABP4 and SLC2A4 was unchanged, while the expected inhibitory effect was seen with TNF. Both cytokines increased the mRNA level of CCL2 and MMP3, which may further promote inflammation in WAT. Thus, the myeloid-restricted nature of CEBPE precludes the expression of RETN in human adipocytes which, however, are targeted by this innate immune-derived proinflammatory cytokine.

## Introduction

Obesity is clearly associated with the development of insulin resistance, diabetes and other metabolic and cardiovascular disorders in man. An important mechanistic link between these debilitating diseases and an increased fat mass is thought to be the anomalous production by adipose cells of a group of diverse effector molecules, collectively named adipocytokines. Like many other assumptions, this hormonal or endocrine hypothesis was primarily explored in mouse, although human genome and physiology significantly diverge from the rodent settings. Recently, mouse resistin was described as a novel obesity-mediated adipocytokine that impairs glucose homeostasis by affecting both insulin-stimulated glucose uptake in adipose tissue and hepatic glucose production during fasting. However, there were initially two opposite views on human resistin. Like in rodents, the human homolog appears as an adipose-specific and obesity-regulated antagonist of insulin action or, conversely, blood/immune cells and not adipocytes are source of resistin.

Mouse resistin, official gene symbol Retn (Table 1), is also known under other aliases depending on how it has been identified. Holcomb *et al.* [1] discovered mouse Fizz3 and human FIZZ3 by searching sequence databases for genes similar to the novel murine gene Fizz1, found in inflammatory zone 1. Kim *et al.* [2] named a new murine protein as solely adipose tissue-specific secretory factor (ADSF) and Steppan *et al.* [3] called it resistin for causing resistance to insulin. The mouse resistin like alpha (Retnla) and beta (Retnlb) genes were also known at that time [1], [4], while one additional gene, resistin like gamma (Retnlg), was described later [5], [6]. So far, only two corresponding human genetic traits have been variably recognized (Table 1), but registered as RETN and RETNLB. These original reports [1]–[3] concordantly highlighted that murine resistin is produced entirely by adipocytes, but the source of human resistin remained unknown.

**Table 1:**
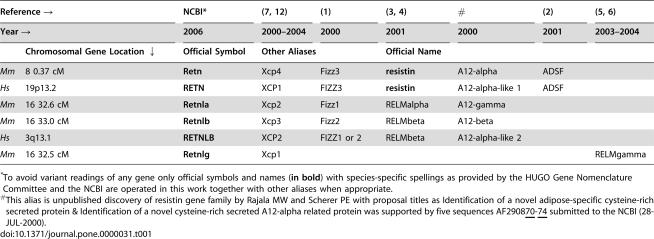
Different Resistin Gene Families in *Mus musculus* (*Mm*) and *Homo sapiens* (*Hs*)

Reference →	NCBI*	(7, 12)	(1)	(3, 4)	#	(2)	(5, 6)
Year →	2006	2000–2004	2000	2001	2000	2001	2003–2004
Chromosomal Gene Location ↓	Official Symbol	Other Aliases		Official Name			
*Mm* 8 0.37 cM	**Retn**	Xcp4	Fizz3	**resistin**	A12-alpha	ADSF	
*Hs* 19p13.2	**RETN**	XCP1	FIZZ3	**resistin**	A12-alpha-like 1	ADSF	
*Mm* 16 32.6 cM	**Retnla**	Xcp2	Fizz1	RELMalpha	A12-gamma		
*Mm* 16 33.0 cM	**Retnlb**	Xcp3	Fizz2	RELMbeta	A12-beta		
*Hs* 3q13.1	**RETNLB**	XCP2	FIZZ1 or 2	RELMbeta	A12-alpha-like 2		
*Mm* 16 32.5 cM	**Retnlg**	Xcp1					RELMgamma

* To avoid variant readings of any gene only official symbols and names (**in bold**) with species-specific spellings as provided by the HUGO Gene Nomenclature Committee and the NCBI are operated in this work together with other aliases when appropriate.

# This alias is unpublished discovery of resistin gene family by Rajala MW and Scherer PE with proposal titles as Identification of a novel adipose-specific cysteine-rich secreted protein & Identification of a novel cysteine-rich secreted A12-alpha related protein was supported by five sequences AF290870-74 submitted to the NCBI (28-JUL-2000).

As frequently happens, the clarification came from another field. The earliest annotation of the mouse Retnlg was provided in accession BE847001 [7]. This novel gene for short cysteine-rich protein 1 (homologous to FIZZ1) was not expressed in myeloid cells from C/EBP-epsilon knockout mice. The myeloid-specific nuclear transcription factor CCAAT/enhancer binding protein epsilon (CEBPE) has been characterized previously [8]. The same group also identified all resistin-related genes, which were named as ten-cysteine-proteins (XCP) for their characteristic C-terminal stretch of 10 cysteine residues with equal spacing (Table 1). Resistin (RETN) was defined in AF352730 as *Homo sapiens* C/EBP-epsilon regulated myeloid-specific secreted cysteine-rich protein precursor (HXCP1) gene. These findings as well as the broadly accepted belief that human resistin, RETN, is homologous to the murine counterpart Retn were tested in our earlier study of the putative role of RETN as a factor involved in insulin resistance [9]. We showed that RETN was readily expressed in monocytes. However, RETN mRNA levels were low and unstably detected in WAT while RETN transcript was totally absent in isolated adipocytes and muscle samples from non-obese euglycemic but insulin-resistant subjects. Therefore we, like others [10], [11], concluded that resistin in WAT and skeletal muscle is unrelated to insulin action in man [9].

Firm evidence of the similarity between mouse resistin like gamma, Retnlg/Xcp1, and human resistin, RETN/XCP1, was recently published by Chumakov *et al.* [12]. Interestingly, the expression of these genes was exclusively found in myeloid cells with the highest level in bone marrow. Their transcription is dependent on mouse Cebpe and human CEBPE [12]. The latter appears vital for terminal differentiation and functional maturation of human committed granulocyte progenitor cells [13]. In both species, this myeloid transcription factor was shown to be essential for, and co-expressed with, XCP1/RETN and Xcp1/Retnlg. Furthermore, its functional disruption in either species results in total silencing of RETN and Retnlg, whereas the induction triggers their expression even in non-myeloid cells [12]. Additionally, released human resistin as well as mouse resistin like gamma proteins play the same role in the chemotaxis of bone marrow-derived myeloid cells and specifically interact with human neutrophil α-defensin [12]. Taken together, these findings indicate that human RETN is the biological analogue of murine Retnlg.

This widely disregarded dualism of human resistin, i.e., RETN gene being a homologue of mouse Retn but a regulatory and functional analogue of murine Retnlg, has led to discrepant and controversial observations. All along, the mixed conception for human resistin as an adipocyte- and macrophage-derived cytokine with metabolic and inflammatory activities was a diffuse and confusing paradigm. As a result, none of the basic investigations and clinical trials could explicitly clarify which human cells produce resistin and its physiological role. Adding to the difficulty, resistin gene lineages have seemingly underwent various alterations during the evolution of different mammalian species. Therefore, in the present work, we tested all resistin regulatory, functional and also emerging evolutionary concepts in different human and mouse models aiming to explain the Resistin Paradox decisively. Overall, in addition to previous studies [9], [14], our new findings strongly support the immune-specific role of human resistin as a significant local and systemic modulator of inflammation.

## Results

### Cell- and tissue-specific expression of human resistin

We first examined if isolated human mature adipose cells, like their murine counterparts, express resistin and/or the necessary transcription factor CEBPE. For this purpose, we studied different leukocytic specimens as positive controls together with human WAT, preadipocytes and muscle as well as murine adipose cells and macrophages (Table 2). Human cells were tested for CEBPE and RETN as well as EMR1 (or murine alias F4/80), a leukocyte-specific receptor which indicates the presence of white blood cells in a sample. The transcription level of CEBPE was readily detectable and RETN was also expressed in freshly prepared PBMC and synoviocytes as well as in differentiated macrophages, albeit at a lower level. In these myeloid samples we observed a clear CEBPE-RETN correlation (R^2^ = 0.4, p<0.006, n = 9) and a lower, but still significant, association between CEBPE and EMR1 (R^2^ = 0.2, p<0.006, n = 9). In contrast, THP-1 monocytes displayed an apparent discrepancy with high expression of CEBPE but not of RETN. Also, PBMC cultured for 24 hours revealed a gradual decline in RETN transcription while the CEBPE mRNA level remained almost unchanged. After the culture period, the expression profiles in PBMC resembled that of THP-1 cells and *in vitro* differentiated macrophages (Table 2). Taken together, these results show that freshly isolated human primary inflammatory cells co-express CEBPE and RETN. However, RETN expression decreases during culture while CEBPE remains more stable.

**Table 2:**
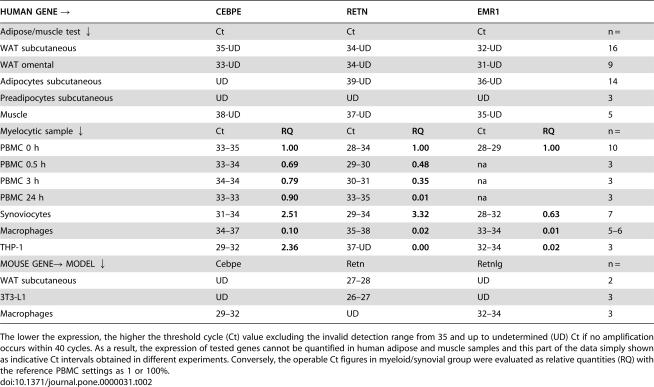
Examination of Gene Expression in Basally Conditioned Tissue and Cell Cultures

HUMAN GENE →	CEBPE		RETN		EMR1		
Adipose/muscle test ↓	Ct		Ct		Ct		n =
WAT subcutaneous	35-UD		34-UD		32-UD		16
WAT omental	33-UD		34-UD		31-UD		9
Adipocytes subcutaneous	UD		39-UD		36-UD		14
Preadipocytes subcutaneous	UD		UD		UD		3
Muscle	38-UD		37-UD		35-UD		5
Myelocytic sample ↓	Ct	**RQ**	Ct	**RQ**	Ct	**RQ**	n =
PBMC 0 h	33–35	**1.00**	28–34	**1.00**	28–29	**1.00**	10
PBMC 0.5 h	33–34	**0.69**	29–30	**0.48**	na		3
PBMC 3 h	34–34	**0.79**	30–31	**0.35**	na		3
PBMC 24 h	33–33	**0.90**	33–35	**0.01**	na		3
Synoviocytes	31–34	**2.51**	29–34	**3.32**	28–32	**0.63**	7
Macrophages	34–37	**0.10**	35–38	**0.02**	33–34	**0.01**	5–6
THP-1	29–32	**2.36**	37-UD	**0.00**	32–34	**0.02**	3
MOUSE GENE→ MODEL ↓	Cebpe		Retn		Retnlg		n =
WAT subcutaneous	UD		27–28		UD		2
3T3-L1	UD		26–27		UD		3
Macrophages	29–32		UD		32–34		3

The lower the expression, the higher the threshold cycle (Ct) value excluding the invalid detection range from 35 and up to undetermined (UD) Ct if no amplification occurs within 40 cycles. As a result, the expression of tested genes cannot be quantified in human adipose and muscle samples and this part of the data simply shown as indicative Ct intervals obtained in different experiments. Conversely, the operable Ct figures in myeloid/synovial group were evaluated as relative quantities (RQ) with the reference PBMC settings as 1 or 100%.

In some freshly obtained *ex vivo* human WAT specimens, CEBPE and RETN were detected with valid Ct values (Table 2). However, their expression was unrelated to fat depot, body mass index (BMI) or insulin resistance. Significantly, CEBPE and RETN mRNA were co-detected with the EMR1 transcript demonstrating that the expression of all these genes is limited to the myeloid cells present in a sample. This was further supported by the finding that homogeneous cell populations of either human preadipocytes or isolated subcutaneous adipocytes exhibited predominantly undetermined Ct for all these genes. Thus, the source of this low and variable expression of CEBPE and RETN in human WAT is likely to be blood leukocytes and/or resident macrophages - all EMR1-positive. Together, these results corroborate that isolated human primary adipocytes and preadipocytes do not express RETN or its transcriptional activator CEBPE.

In contrast to these findings, Cebpe expression in mouse peritoneal macrophages was co-localized with Retnlg, but not with Retn transcription; the latter being solely restricted to adipose specimens (Table 2). The basis of such disparity between these two mammalian species, i.e., the differences seen between orthologous resistin genes, combined with the obvious similarity of non-homologous RETN and Retnlg, is presently unclear.

### Re-examination of RETN expression by specifically selected TaqMan real-time PCR assays

It is important to clarify the reason for the controversial results produced by different authors regarding the expression of RETN in human adipose samples using the same real-time RT-PCR technique (see e.g., 9, 10 versus 11, 15, 16). Until recently, there was no inter-laboratory reference test available, but we have now compared one of three standard RETN Gene Expression Assays developed by Applied Biosystems with other resistin TaqMan constructs (Table 3) all-in-one-test. We purposely used the absolute quantification or the standard curve method because only this procedure firmly generates several key indices. The correlation coefficient represents the accuracy of the standard curve where a slope value around −3.5 means the highest PCR efficiency (Table 4, Panel A). Together, they indicate the reliability of each assay and also qualify the comparison between them (Table 4, Panel B).

**Table 3:**
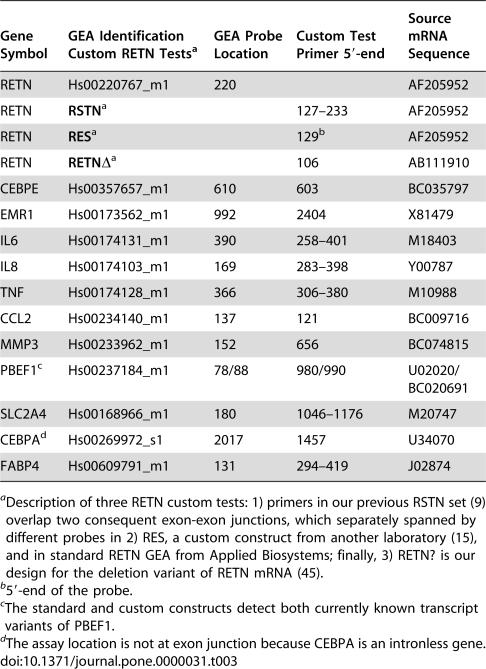
Common Principle of the Over-Exon-Boundary Location of Probe in Reference Gene Expression Assays (GEA) and Primer(s) in our Custom Tests

Gene Symbol	GEA Identification Custom RETN Tests^a^	GEA Probe Location	Custom Test Primer 5′-end	Source mRNA Sequence
RETN	Hs00220767_m1	220		AF205952
RETN	**RSTN** a		127–233	AF205952
RETN	**RES** a		129^b^	AF205952
RETN	**RETN** *Δ* a		106	AB111910
CEBPE	Hs00357657_m1	610	603	BC035797
EMR1	Hs00173562_m1	992	2404	X81479
IL6	Hs00174131_m1	390	258–401	M18403
IL8	Hs00174103_m1	169	283–398	Y00787
TNF	Hs00174128_m1	366	306–380	M10988
CCL2	Hs00234140_m1	137	121	BC009716
MMP3	Hs00233962_m1	152	656	BC074815
PBEF1^c^	Hs00237184_m1	78/88	980/990	U02020/BC020691
SLC2A4	Hs00168966_m1	180	1046–1176	M20747
CEBPA^d^	Hs00269972_s1	2017	1457	U34070
FABP4	Hs00609791_m1	131	294–419	J02874

a Description of three RETN custom tests: 1) primers in our previous RSTN set (9) overlap two consequent exon-exon junctions, which separately spanned by different probes in 2) RES, a custom construct from another laboratory (15), and in standard RETN GEA from Applied Biosystems; finally, 3) RETN? is our design for the deletion variant of RETN mRNA (45).

b 5′-end of the probe.

c The standard and custom constructs detect both currently known transcript variants of PBEF1.

d The assay location is not at exon junction because CEBPA is an intronless gene.

**Table 4:**
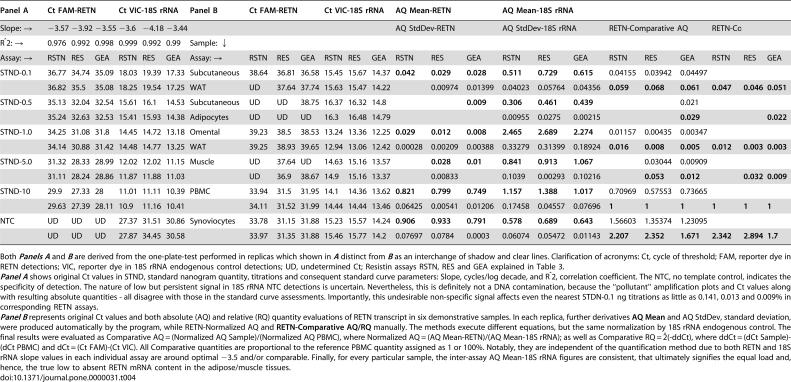
Special Evaluation of Different RETN TaqMan Assays by Absolute (AQ) and Relative (RQ) Quantification Analyses

Panel A	Ct FAM-RETN			Ct VIC-18S rRNA			Panel B	Ct FAM-RETN			Ct VIC-18S rRNA			AQ Mean-RETN			AQ Mean-18S rRNA			RETN-Normalised AQ					
Slope: →	−3.57	−3.92	−3.55	−3.6	−4.18	−3.44								AQ StdDev-RETN			AQ StdDev-18S rRNA			RETN-Comparative AQ			RETN-Comparative RQ		
R̂2: →	0.976	0.992	0.998	0.999	0.992	0.99	Sample: ↓																		
Assay: →	RSTN	RES	GEA	RSTN	RES	GEA	Assay: →	RSTN	RES	GEA	RSTN	RES	GEA	RSTN	RES	GEA	RSTN	RES	GEA	RSTN	RES	GEA	RSTN	RES	GEA
STND-0.1	36.77	34.74	35.09	18.03	19.39	17.33	Subcutaneous	38.64	36.81	36.58	15.45	15.67	14.37	**0.042**	**0.029**	**0.028**	**0.511**	**0.729**	**0.615**	0.04155	0.03942	0.04497			
	36.82	35.5	35.08	18.25	19.54	17.25	WAT	UD	37.64	37.74	15.63	15.47	14.22		0.00974	0.01399	0.04023	0.05764	0.04356	**0.059**	**0.068**	**0.061**	**0.047**	**0.046**	**0.051**
STND-0.5	35.13	32.04	32.54	15.61	16.1	14.53	Subcutaneous	UD	UD	38.75	16.37	16.32	14.8			**0.009**	**0.306**	**0.461**	**0.439**			0.021			
	35.24	32.63	32.53	15.41	15.93	14.38	Adipocytes	UD	UD	UD	16.3	16.48	14.79				0.00955	0.0275	0.00215			**0.029**			**0.022**
STND-1.0	34.25	31.08	31.8	14.45	14.72	13.18	Omental	39.23	38.5	38.53	13.24	13.36	12.25	**0.029**	**0.012**	**0.008**	**2.465**	**2.689**	**2.274**	0.01157	0.00435	0.00347			
	34.14	30.88	31.42	14.48	14.77	13.25	WAT	39.25	38.93	39.65	12.94	13.06	12.42	0.00028	0.00209	0.00388	0.33279	0.31399	0.18924	**0.016**	**0.008**	**0.005**	**0.012**	**0.003**	**0.003**
STND-5.0	31.32	28.33	28.99	12.02	12.02	11.15	Muscle	UD	37.64	UD	14.63	15.16	13.57		**0.028**	**0.01**	**0.841**	**0.913**	**1.067**		0.03044	0.00909			
	31.11	28.24	28.86	11.87	11.88	11.03		UD	36.9	38.67	14.9	15.16	13.37		0.00833		0.1039	0.00293	0.10216		**0.053**	**0.012**		**0.032**	**0.009**
STND-10	29.9	27.33	28	11.01	11.11	10.39	PBMC	33.94	31.5	31.95	14.1	14.36	13.62	**0.821**	**0.799**	**0.749**	**1.157**	**1.388**	**1.017**	0.70969	0.57553	0.73665			
	29.63	27.39	28.11	10.9	11.16	10.41		34.11	31.52	31.99	14.44	14.44	13.46	0.06425	0.00541	0.01206	0.17458	0.04557	0.07696	**1**	**1**	**1**	**1**	**1**	**1**
NTC	UD	UD	UD	27.37	31.51	30.86	Synoviocytes	33.78	31.15	31.88	15.23	15.57	14.24	**0.906**	**0.933**	**0.791**	**0.578**	**0.689**	**0.643**	1.56603	1.35374	1.23095			
	UD	UD	UD	27.87	34.45	30.58		33.97	31.35	31.88	15.46	15.77	14.2	0.07697	0.0784	0.0003	0.06074	0.05472	0.01143	**2.207**	**2.352**	**1.671**	**2.342**	**2.894**	**1.7**

Both ***Panels A*** and ***B*** are derived from the one-plate-test performed in replicas which shown in ***A*** distinct from ***B*** as an interchange of shadow and clear lines. Clarification of acronyms: Ct, cycle of threshold; FAM, reporter dye in RETN detections; VIC, reporter dye in 18S rRNA endogenous control detections; UD, undetermined Ct; Resistin assays RSTN, RES and GEA explained in Table 3.

***Panel A*** shows original Ct values in STND, standard nanogram quantity, titrations and consequent standard curve parameters: Slope, cycles/log decade, and R̂2, correlation coefficient. The NTC, no template control, indicates the specificity of detection. The nature of low but persistent signal in 18S rRNA NTC detections is uncertain. Nevertheless, this is definitely not a DNA contamination, because the “pollutant” amplification plots and Ct values along with resulting absolute quantities - all disagree with those in the standard curve assessments. Importantly, this undesirable non-specific signal affects even the nearest STDN-0.1 ng titrations as little as 0.141, 0.013 and 0.009% in corresponding RETN assays.

***Panel B*** represents original Ct values and both absolute (AQ) and relative (RQ) quantity evaluations of RETN transcript in six demonstrative samples. In each replica, further derivatives **AQ Mean** and AQ StdDev, standard deviation, were produced automatically by the program, while RETN-Normalized AQ and **RETN-Comparative AQ/RQ** manually. The methods execute different equations, but the same normalization by 18S rRNA endogenous control. The final results were evaluated as Comparative AQ = (Normalized AQ Sample)/(Normalized AQ PBMC), where Normalized AQ = (AQ Mean-RETN)/(AQ Mean-18S rRNA); as well as Comparative RQ = 2̂(-ddCt), where ddCt = (dCt Sample)-(dCt PBMC) and dCt = (Ct FAM)-(Ct VIC). All Comparative quantities are proportional to the reference PBMC quantity assigned as 1 or 100%. Notably, they are independent of the quantification method due to both RETN and 18S rRNA slope values in each individual assay are around optimal −3.5 and/or comparable. Finally, for every particular sample, the inter-assay AQ Mean-18S rRNA figures are consistent, that ultimately signifies the equal load and, hence, the true low to absent RETN mRNA content in the adipose/muscle tissues.

In general, all three FAM/VIC multiplexed assays have similar detection effectiveness, while the fluctuations among Ct and slope numerals (Table 4) are mostly attributable to the differences in threshold and in ordinary set design including specific physico-chemical qualities of the probe (see Methods and Table 3). The data show that all assays are unable to detect RETN transcript in isolated adipocytes (Table 4, Panel B). Likewise, they cannot quantify it correctly in WAT and skeletal muscle even if Ct FAM values were determined. These values are within the invalid detection range from 35 to undetermined and significantly scattered in a replica in contrast to the consistent Ct figures in the blood and synovial samples. As a result, both inter-assay and intra-replica variations are small (6.5 and 4.7%) in the myeloid analyses versus the gross deviations (68 and 32%) in the adipose-muscle assessments. The intra-assay endogenous 18S rRNA control is also the inter-assay reference demonstrating a modest 8.4% divergence between all replicas, samples and assays. Thus, the complete uniformity of the trial confirms the low to absent detection level of RETN in human adipose tissue. In contrast, synoviocytes from patients with rheumatoid arthritis show that RETN expression is elevated at a site of inflammation (Tables 2 and 4).

We also investigated the potential expression of an alternatively spliced variant of RETN with deletion of the entire exon 3 (RETNΔ in Table 3) in the same one-plate-test. The copy number of this molecule was extremely low with an approximate ratio of 1∶240 of the truncated vs. full-length transcripts. This was only possible to evaluate in PBMC (data not shown).

Altogether, these results demonstrate that neither alternative splicing nor variations between real-time PCR assays, but rather the high sensitivity of the method accounted for the detection of even a few RETN-positive leukocytes, either peripheral blood cells and/or the tissue-residing inflammatory cells, which were present in some human WAT samples.

### Proinflammatory effects of resistin in human adipose tissue

**Figure 1:**
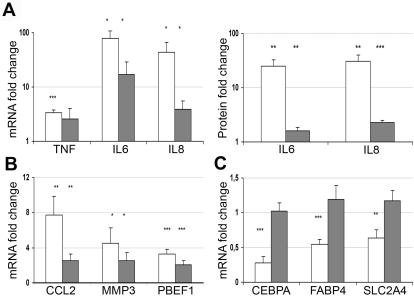
Resistin Preferentially Induces Inflammatory Markers in Human WAT. Human WAT explants were incubated with 10 ng/ml TNF, white bars, or 50 ng/ml resistin, grey bars, for 24 hrs and the fold change for several markers assessed by comparison with their level in basal cultures after 24 hrs. In ***A*** both gene (left) and protein (right) expressions of proinflammatory cytokines are shown, while ***B*** represents other inflammation-related effects in WAT. ***C*** demonstrates different effects of TNF and resistin on markers of adipocyte differentiation. *, *P*<0.1; **, *P*<0.01; ***, *P*<0.001.

As concluded above, resistin is not expressed in human primary adipocytes but it is present in immune cells which are found in subcutaneous WAT in obesity [17] and also infiltrate epicardial fat depot in patients with coronary disease independent of BMI and diabetes [18]. Consequently, human adipocytes may be target cells for resistin. We investigated this possibility by exposing biopsies of human WAT to a physiologically relevant [14] concentration of resistin (50 ng/ml) or, as a positive control, 10 ng/ml of TNF for 24 hrs. As shown in Figure 1A (left), both cytokines induced expression of several inflammatory molecules, although to a different extent. TNF enhanced the expression of IL6 and IL8 79- and 44-fold, respectively, and its own gene about 3.4-fold. Similarly, secretion of both IL6 and IL8 to the culture medium was increased on average 28-times (Figure 1A, right). Resistin also elevated both the mRNA level (17- and 4-fold) and protein secretion (1.6- and 2.3-fold) of IL6 and IL8, while only a small (2.6-fold) and insignificant stimulatory effect was seen on the TNF gene (Figure 1A, left). Additionally, we found that resistin, similar to TNF, induces the CCL2, MMP3 and PBEF1 genes, which are all relevant to the inflammatory process in the adipose tissue (Figure. 1B). Although both TNF and RETN exerted a proinflammatory action on the WAT explants, their effects on the markers of adipose differentiation varied. As expected, TNF reduced the expression of CEBPA, FABP4 and SLC2A4 (GLUT4) by 40–70% while these genes remained unchanged upon exposure to resistin at this concentration (Figure. 1C). Together, these findings show that human adipocytes do not produce resistin but they are target cells for this cytokine which, in analogy with TNF, enhances the inflammatory process in adipose tissue.

## Discussion

Experimental human data regarding the nature of RETN-expressing cells as well as the physiological role of secreted resistin are controversial. In contrast to our own studies [9] and also those from several other laboratories [10], [12], [19]–[24], McTernan *et al.* [16] reported that resistin mRNA and protein could be recovered from both isolated human pre-adipocytes and mature adipose cells. Moreover, it was stated that RETN is differentially expressed between various fat depots [15]. In both studies, a higher signal was found in abdominal WAT suggesting that, similar to rodents, resistin may connect central obesity to insulin resistance and diabetes in man. Also, Degawa-Yamauchi *et al.* [25] detected RETN protein in isolated adipocytes and observed a positive association between serum resistin level and adiposity. Finally, in the study by Janke *et al.* [11], human preadipocyte differentiation was coupled with a declining gene expression. Although this observation was corroborated by barely measurable mRNA levels in primary cultured mature adipocytes, no link with insulin resistance was found.

In contrast to these discrepant results, various investigations in man consistently revealed the presence of resistin in myeloid cells: both primary monocyte-derived and/or differentiated macrophages [9], [10], [19]–[21], [26]–[28] as well as in primary acute leukemia cells and the myelocytic lines U937 and HL60 [22]. This leukocyte expression of human resistin was recently discovered to depend on the myeloid-specific nuclear transcription factor CEBPE [12]. The action of CEBPE was previously known to be restricted to the haematopoietic tissues, especially myeloid granulocyte/macrophage and lymphoid T-cell lineages in the bone marrow [8], [13], [29], [30]. In line with, but independent of the CEBPE research, the highest level of resistin was found in the bone marrow [20], [24]. Moreover, human resistin possesses diverse immune activities such as induction of chemotaxis of myeloid cells [12], as an inflammatory cytokine in rheumatoid arthritis [14] and in endotoxemia [26], and as a macrophage-derived atherogenic factor inducing endothelial dysfunction and vascular smooth muscle cell migration [27].

Human RETN and mouse Retn genes are orthologs [22] and, consequently, due to their evolutionary genetic relatedness, thought to be homologous traits. Surprisingly, human resistin exhibits characteristics which are clearly distinct from those of its mouse counterpart Retn. Furthermore, RETN shares prominent similarity with Retnlg [12], another resistin family member identified only in rodents. This still unrecognized phenomenon took place during evolution of other mammals, like cow and pig, also expressing RETN in myeloid cells [31] rather than in adipose tissue [31], [32]. The current understanding of this discrepancy is difficult since mouse and human resistin, nevertheless, exhibit common arthritogenic [14] and atherogenic [33], [34] features even if they are produced by different cells.

In the present study, we carefully examined in which human cells resistin is expressed and also focused on the CEBPE-RETN transcriptional link and the putative immune/inflammatory action in WAT. Consistent with Chumakov *et al.* [12], we found that RETN generally follows CEBPE expression. Both transcripts were also determined to be attributes of myeloid cells because of the stringent co-localization with EMR1, which appeared to be a common leukocytic marker (Table 2) rather than an exclusive macrophage determinant [35]. Therefore, it is essential to further define the precise mechanism of RETN expression in particular types of leukocytes present in blood and synovia as well as in adipose tissue. We observed a clear discrepancy in expression of the RETN and CEBPE genes in myeloid samples newly obtained *in vivo* vs. the same cells conditioned *in vitro*. There was a high and concomitant CEBPE-RETN expression in fresh blood and synovial fluid specimens but a reduced and dissociated transcription in cultured PBMC, macrophages and THP-1 cells. The latter immortalized cells do not express RETN but visibly display CEBPE as also corroborated by Northern blot analysis [12], [13]. Nonetheless, very low CEBPE expression in macrophages, seen here and by others [13], was yet accompanied by RETN transcription, albeit at a low level (Table 2). Although the RETN expression is under ultimate transcriptional control of CEBPE [12], the activity of CEBPE is modified by phosphorylation [36], heterodimerization with abundant associate proteins [37] and a synergistic effect of MYB and ATF4 [12]. Other transcriptional factors, like PPAR gamma [20], SP1 and SP3 [38] and also SREBF1 [39], could influence the RETN regulation *in vitro*. The role and activation of CEBPE is different during granulocytic and monocytic differentiation [13]. Consequently, CEBPE-to-RETN regulatory strength can change considerably upon various conditions and in certain cell type (Table 2). Our results show a higher expression level of both genes in synoviocytes, presumably granulocytes, than in peripheral monocytes as well as a maintained CEBPE-RETN proportion in these *ex vivo* models.

Importantly, human preadipocytes and isolated fat cells were clearly devoid of RETN mRNA (Tables 2 and 4), similar to endothelial cells, smooth muscle and many other non-myeloid tissues, which have been tested [9], [10], [12], [19]–[24], [27], [28]. Furthermore, the resistin release by adipose tissue is not due to adipose cells [28], [40]. Together, these data suggest that human resistin should not be acknowledged as an adipose-secreted hormone but as a specific myeloid-derived cytokine.

We also studied the murine genes Cebpe, Retn and Retnlg, because the latter was found as a regulatory and functional equivalent of human RETN [12]. Only Retn was detected in adipose tissue of ob/ob mice and 3T3-L1 cells, whereas, in agreement with other results [5]–[7], [12], Cebpe and Retnlg expression was observed in mouse peritoneal macrophages. However, very little is known about similarities and differences between rodent Retnlg and human RETN since only one study exists in the field [12]. Obviously, lack of recognition of this fact is the basis of a recent proposal of murine Retnlg and the non-existent human RETNLG (Table 1) as a hormonal link between the digestive tract and insulin resistance in both species [41]. Such conclusions only create confusion. Also a presumed arthritogenic Retn-Retnlg cross-function in mice [14] needs a further clarification.

Despite many RETN mRNA evaluations by real-time PCR, different problems related to bioinformatic errors and test designs exist [23], [24]. In addition, conflicting Northern and SYBR Green RT-PCR results [22] and lack of initial Ct values in most reports make firm conclusions impossible. With the aim of having a gene expression analysis as decisive as possible, we included the impartial inter-laboratory reference TaqMan RT-PCR assays from Applied Biosystems in parallel to our custom tests (Table 3). They all demonstrated comparable outcomes over the entire investigation also in the single-shot trial of three RETN assays (Table 4), including the one that was reported to be successful in the quantification of resistin mRNA in human adipocytes [15], [16]. All three constructs detect a common sequence in full-length RETN mRNA (Table 3) and the multiplexed assays have similar detection efficiency (Table 4). A higher level of RETN transcript was detected in inflamed synoviocytes in comparison to normal PBMC. In contrast, no valid quantification could be made in adipose and muscle samples by any of these optimized tests, as indicated by both slope and R^2^ values in Table 4. To obtain a RETN signal in the adipose analysis, McTernan *et al.* [15], [16], [42], [43] overloaded the cDNA to 115 ng in which a proportion of 18S ribosomal RNA cannot be linearly distinguished and, hence, the Ct of the internal reference can not normalize the Ct of the tested gene. Under these conditions, the dCt figures and the final quantity evaluations are, at best, conjectural [15], [16], [42], [43]. Taken together, we conclude that RETN is not expressed in human isolated fat cells and that reports claiming positive results have methodological shortcomings which have led to spurious conclusions.

A proinflammatory nature of resistin has been suggested, but its precise action in hepatocytes [28] and adipose tissue [24] is unclear. We previously noticed that, in PBMC, resistin can induce both gene expression and secretion of inflammatory cytokines like IL6, IL8 and TNF of which only TNF, but not resistin, rapidly up-regulates RETN [14]. Interestingly, lipopolysaccharide, a bacterial endotoxin, controls RETN in the same, presumably TNF-mediated, manner (unpublished data). Here we demonstrate that, like in PBMC, resistin can also enhance the gene and protein expression of IL6 and IL8 in human WAT, while the effect on TNF mRNA expression was small. However, in contrast to the well-known TNF-induced suppression of different adipose-specific markers such as CEBPA, FABP4 and SLC2A4, these genes were not affected by resistin at the concentration of 50 ng/ml. These data concur with the unchanged insulin-stimulated glucose uptake by human differentiated adipocytes treated with high concentrations of recombinant resistin (58.5–5850 ng/ml) [24]. However, McTernan *et al.* [42] reported that 0.1–50 ng of resistin impaired the glucose incorporation into adipocytes. The cause for this discrepancy is unclear.

We also found that resistin, similar to TNF, induces other proinflammatory genes in human adipose tissue. Chemokine C-C motif ligand 2 (CCL2) is a cytokine that displays chemotactic activity for monocytes. Matrix metallopeptidase 3 (MMP3) may affect the extracellular milieu of adipose tissue akin to that in arthritis. Also PBEF1, pre-B-cell colony enhancing factor 1 or visfatin, was activated by these cytokines. Both the basal and resistin-stimulated gene expression profiles in WAT cultures bear a remarkable resemblance to that seen in PBMC [14], but with clear cell-type differences.

In conclusion, human fat cells do not express or secrete RETN but WAT is evidently a target tissue for resistin. This finding, together with the effect of resistin on PBMC, makes it clear that resistin is an immune-derived systemic and locally acting proinflammatory cytokine in man. However, in contrast to TNF, it does not induce a rapid “dedifferentiation” of the adipose cells. This variation is intriguing and obviously implicates that the resistin intracellular signaling pathway is distinct from that of TNF although both activate NFκB.

## Materials and Methods

### Source of RNA and culture procedures

All biopsy procedures were approved by the Ethical Committee of the Göteborg University. Biopsies of human abdominal subcutaneous adipose tissue were obtained from surgical operations for nonsystemic abdominal disorders (mainly uterine extirpation, gall bladder disease) from seven otherwise healthy and nonobese females (BMI 26.1±1.8 kg/m2). The biopsies were cleaned of blood and tissue debris, dissected into 10–20 mg explants and incubated in medium 199 HANKS containing 1% human albumin, 10mM HEPES, and 5.6 mM glucose according to previously reported procedures [44]. WAT explants were cultured with 10 ng/ml TNF (Sigma, UK) or 50 ng/ml human recombinant RETN (PeproTech EC, UK) or without additives for 24 h. The medium concentrations of the different cytokines were measured with ELISA (R&D Systems, Minneapolis, MN, USA and BioVendor, Czech Republic).

Preadipocytes were also obtained from the stromal vascular fraction of biopsies of the abdominal subcutaneous WAT of non-obese individuals. Another set of both omental and subcutaneous adipose tissue biopsies were collected during surgical excision of non-disseminated gastrointestinal cancers from 9 individuals (BMI 26.0±1.2 kg/m2) for the extraction of total RNA. PBMC were isolated and stimulated by resistin and LPS as described [14]. The PBMC fraction from a Lymphoprep density gradient consists of mononuclear leukocytes, lymphocytes and less than 1% of polymorphonuclear granulocytes. Synovial fluids from patients with rheumatoid arthritis were centrifuged at 3000×g for 10 minutes to pool monocytes, granulocytes, fibroblasts and other cells collectively called synoviocytes. The monocytic cell line, THP-1, was used as a standard of CEBPE, RETN and EMR1 gene expression at basal conditions. The RNA from human macrophages was kindly provided by Dr. B. Staels (Lille, France). Murine macrophages were acquired from peritoneal exudates of BALB-C mice. Additional human skeletal muscle and isolated mature adipocytes, also 3T3-L1 and mouse WAT samples have been explained in past studies [9], [44].

### Real-time RT-PCR

All cellular samples were immediately subjected to the lysis buffer and further processed with RNA extraction according to the RNeasy Mini Kit manual (Qiagen, UK). Tissue specimens were placed in RNA Later (Ambion, Austin, TX, USA) prior to a cell disintegration by guanidinium thiocyanate followed by chloroform extraction and final RNA purification via CsCl gradient ultracentrifugation as described earlier [9]. Total RNA was obtained from tissue and cell samples before and after culture. RNA concentration and λ 260/280 nm ratio was assessed and complementary DNA synthesized according to the TaqMan® Reverse Transcription Reagents kit manual (Applied Biosystems, Foster City, CA, USA) and loaded in each well at an amount approximately equal to 5 ng total RNA. The gene expression was measured with real-time RT-PCR (TaqMan™, Applied Biosystems, Foster City, CA, USA) as previously described in detail [9]. Each custom TaqMan set, if possible, spans one or two exon boundaries in order to facilitate both specificity of RNA detection and efficiency of PCR (Table 3). Sequences were manually designed and checked by means of the PrimerExpress™ program (Applied Biosystems) for the following parameters: secondary structure, melting temperature, GC content, primer-primer and primer-probe dimers. The NCBI GenBank database was used to avoid all known sites of nucleotide polymorphism and unspecific annealing of any oligonucleotide. Table 3 shows the PCR primer positions in source mRNA sequences, which are close to the exon junctions we used in our design. The indicated location in the TaqMan® Gene Expression Assay from Applied Biosystems is exon boundary which is typically situated around the middle of the TaqMan probe. Our custom sequences are available on request.

The real-time PCR tests were run as multiplexed, with simultaneous detection in each well of both target transcript and 18S rRNA, an eukaryotic endogenous control, obtainable as TaqMan® Pre-Developed Assay Reagents (PDAR) for Gene Expression, Applied Biosystems. Due to over 90% abundance of 18S rRNA in total cell RNA, the 18S rRNA PDAR has the primer concentration limited from ordinary 900 nM to 150 nM with the aim to delay very early amplification and to balance two parallel polymerase chain reactions in a multiplexed assay. Nevertheless, as small amounts of total RNA as 1–5 ng produce “premature”, lesser 15, Ct values that are within the method's default background interval from cycle 3 to 15. Therefore, we optimized (Table 4, Panel A) two PDARs specifically pre-mixed as reporter VIC® dye-labeled TaqMan®, either; 1) non-fluorescent quencher MGB, for minor groove binding, or; 2) fluorescent quencher TAMRA™ probes, each at restricted concentration 50 nM, with a pair of unlabeled PCR primers, each further limited to 30 nM in the reaction. The PDARs were, respectively, combined with; 1) TaqMan® Gene Expression Assay at the concentration of 250 nM for FAM-MGB probe and 900 nM of each primer, and; 2) custom constructs formulated as reporter FAM dye-labeled TAMRA dye-quencher probe at 200 nM and both primers at 400 nM final concentration. Comparative Ct and standard curve methods were applied in that order for relative and absolute quantifications but all results were presented as a fold change over the reference level assessed as 1 or 100%.

*Statistical analyses* were carried out with Student's t-test and *P*-values of <0.05 were considered significant.
